# Revisiting CCL-type chemokines in breast cancer and its milieu: prominent targetable chemokines, CCL8 and CCL21

**DOI:** 10.1042/BSR20210033

**Published:** 2021-07-01

**Authors:** Nese Unver

**Affiliations:** Department of Stem Cell Sciences, Graduate School of Health Sciences, Center for Stem Cell Research and Development, Hacettepe University, Ankara, Turkey

**Keywords:** bioinformatics, biomarkers, breast cancers, chemokines, immunology, transcriptomics

## Abstract

The patterns of chemokine expression play a decisive role in both breast cancer prognosis and metastasis. In a recent article published in *Bioscience Reports*, ‘Bioinformatics identification of CCL8/21 as potential prognostic biomarkers in breast cancer microenvironment’, Chen et al. presented that expression of both CCL8 and CCL21 among CCL-type chemokines is prominent for prognosis of the breast cancer, metastasis and chemoresistance (*Biosci Rep* (2020) **40**(11); DOI: 10.1042/BSR20202042). Identifying the sources of the CCL8 and CCL21 in the tumor microenvironment and developing targeting strategies for these chemokines to prevent tumor growth will improve both prognosis and therapeutic outcomes.

## Commentary

The study performed by Chen et al. represents a comprehensive analysis of CCL-type chemokines in breast cancer based on multiple databases [1]. CCL8 and CCL21, which have been demonstrated as potential prognostic markers, were revealed to be co-regulated and associated with leukocyte chemotaxis in the functional enrichment analyses. Also, high CCL8 expression was remarkably associated with negative Progesterone Receptor, negative Estrogen Receptor and Triple-negative breast cancer subgroup [1]. According to another study in the literature, CCL8 expression was not found to be higher in breast cancer compared with normal tissues, but was associated with poorer overall survival (OS) and relapse-free survival (RFS). Moreover, CCL8 was reported to stimulate fibroblasts in the triple-negative breast cancer subgroup. It has also been emphasized that CCL8 expression in these tissues is associated with increased metastatic relapse [2]. The preservation of the CCL8 chemokine gradient in breast cancer epithelial cells and stroma may be critical in metastasis. Furthermore, modification of CCL8 activity influences tumor histopathology and promotes intravasation, extravasation and metastatic processes [3]. Thus, CCL8 contributes to the development of breast cancer by re-shaping the microenvironment with both autocrine and paracrine effects.

CCL8 is not only a chemokine associated with breast cancer, but also undergoes expression changes during the process of mammary gland involution, which is a physiological process. CCL8 is highly expressed during mammary gland evolution compared with puberty, pregnancy and lactation. The high CCL8 expression levels during the involution process of the mammary gland augments the infiltration of pro-tumoral-type (M2-type) macrophages in the second phase of the involution. In this way, CCL8 is a chemoattractant in localizing M2 macrophages with the tumor microenvironment and contributes to the promotion of involution-related breast cancer with its increased expression. Cancer cell vaccination studies in animals with CCL8 deficiency have reported that CCL8 accelerates tumor initiation during involution, but no tumor-promoting effect was detected in nulliparous animals [4]. Consequently, physiological process, histopathologic evaluation as well as molecular subgroup parameters should be also considered in the analysis and interpretation of CCL8 expression levels in breast cancer.

According to Chen et al.’s study, another chemokine that is prominent in breast carcinogenesis is CCL21, which correlates with CCL8 and has the same transcriptional regulators [1]. The overall impact of CCL21 on breast tumor progression can display opposite effects depending on the cell population in which CCL21 and CCR7 receptor are expressed. For instance, CCL21 expression activates the CCR7 receptor by promoting dendritic cells (DCs) and T-cell maturation. CCL21 inhibits tumor growth and enhances survival by CD3 T lymphocytes, particularly in cancer cells with CCR7 expression in mammary tumor-bearing mice. Therefore, CCL21 is known to improve immunogenicity in breast cancer in tandem with its receptor CCR7 [5].

Pro-inflammatory chemokines/cytokines induce the infiltration and activation of antigen-presenting cells. One of the modeling studies on breast cancer treatment was the intra-tumoral administration of (CCL21) and interferon γ (IFN-γ) combination to enhance tumor specific T-cell recruitment in the breast cancer microenvironment. In this way, it can be aimed to increase the immune response activity through pre-primed T cells [6]. On the other hand, CCL21 expression is associated with lymphogenesis and metastasis [7]. In human breast cancer cells, the stimulation of intracellular actin polymerization by CCL21 increases the motility of cancer cells [8] and thus favors metastasis. Epithelial–mesenchymal transition phenotype was also determined during stimulation of breast cancer cell lines HCC1428, MCF-7 and MDA-MB-231 with CCL21. Moreover, it was determined that E-cadherin expression was decreased and Slug, Vimentin and N-cadherin levels were increased *in vitro* [9].

**Figure 1:**
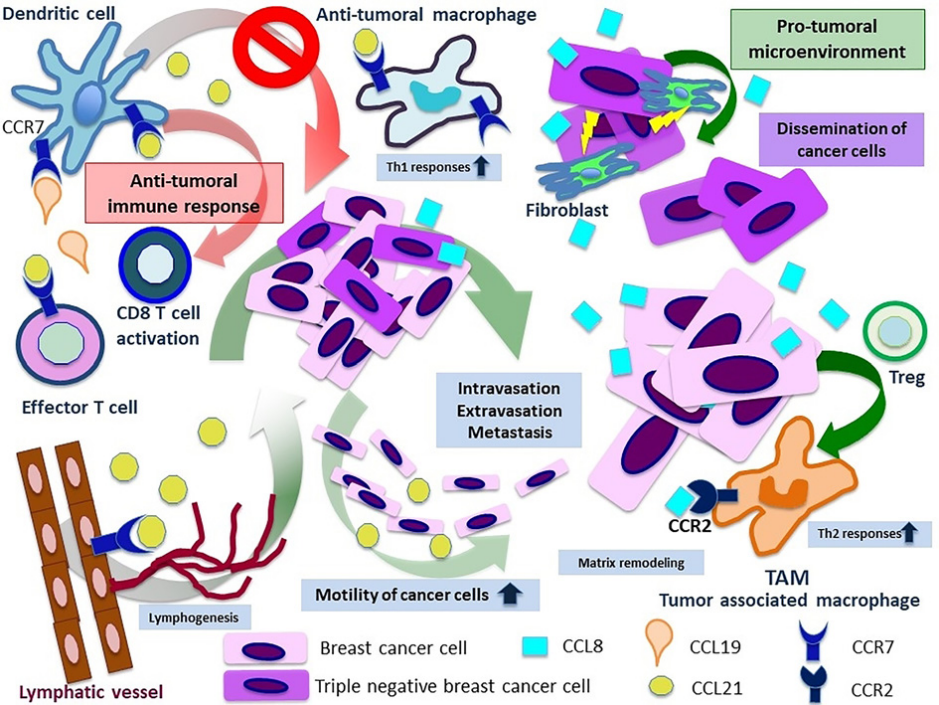
Schematic illustration of the CCL8/CCL21-mediated cross-talk in the breast cancer microenvironment

Tumor cells increase VEGF-C expression in response to the CCL21/CCR7 signal. In lymphatic endothelial cells, the formation of new lymphatic vessels is triggered by VEGFR-3 in response to this CCL21/CCR7 signal axis [10]. According to The Cancer Genome Atlas (TCGA) database, analyses on breast cancer shows that CCL21 expression is positively correlated with T cells. However, it was concluded that CCL21 expression was not associated with macrophage infiltration [11]. Tumor promoting or inhibitory effects of CCL8/CCL21 have been summarized in Figure 1.

As shown in Chen et al.’s study [1], the determination of chemokine panels by stratification according to specific subgroups of breast cancer population using multiple genomic or proteomic datasets constitutes an important basis for validation studies. Identifying the sources of chemokines, CCL8 and CCL21 is critical for both targeting cancer cells and particularly modifying stromal and immune cells via tumor inhibitory mechanisms. Thus, cancer treatment strategies will become more effective in targeting especially aggressive triple-negative breast cancer cells.
